# Enhanced Thermostability and Catalytic Efficiency of Alginate Lyase Alyw203 by Hydrogen Bond Network Reconstruction

**DOI:** 10.3390/md24010006

**Published:** 2025-12-22

**Authors:** Chengcheng Jiang, Jing-Run Ye, Tian-Tian Zhu, Qin Wang, Yan Ma, Zhi-Peng Wang, Chuan-Yang Shi, Ying Wang, Shou-Fu Zhang, Tian-Hong Liu, Hai-Ying Wang

**Affiliations:** 1 Key Laboratory of Sustainable, Development of Polar Fishery, Ministry of Agriculture and Rural Affairs, Yellow Sea Fisheries Research Institute, Chinese Academy of Fishery Sciences, Qingdao 266071, China; jiangcc@ysfri.ac.cn (C.J.); ztt010916@163.com (T.-T.Z.); 2 School of Marine Science and Engineering, Qingdao Agricultural University, Qingdao 266109, China; 3 Qingdao Institute of Marine Bioresources for Nutrition & Health Innovation, Qingdao 266106, China; 4 College of Food Science and Engineering, Ocean University of China, Qingdao 266003, China; 5 Marine Science Research Institute of Shandong Province, Qingdao 266104, China; 6 XingNong WoYe (Shandong) Ecological Agriculture Development Co., Ltd., Binzhou 251999, China

**Keywords:** alginate lyase, site-specific mutation, enzyme activity, thermal stability, hydrogen bond

## Abstract

Alginate lyases are commonly employed for producing alginate oligosaccharides (AOS), but their industrial application is often constrained by low thermal stability and catalytic efficiency. This study engineered mutants of alginate lyase Alyw203 from marine *Vibrio* based on B-factor values and negative ΔΔG values. The L172V mutant exhibited a 2.43-fold increase in half-life at 40 °C, reduced *K*_m_ (from 107 to 65 mg/mL), and enhanced *k*_cat_/*K*_m_ (from 0.07 to 0.35 mL/mg/s), indicating improved thermal stability, substrate affinity, and catalytic efficiency. Molecular dynamics simulations revealed that these improvements originated from reconstructed hydrogen bond networks, which stabilized enzyme–substrate interactions and reduced conformational flexibility. These results demonstrate that rational design focused on strengthening hydrogen bonding can simultaneously improve both stability and activity, offering a promising strategy for industrial AOS production.

## 1. Introduction

Brown-algae-derived alginate, a structurally intricate anionic polysaccharide composed of alternating β-D-mannuronate (M) and α-L-guluronate (G) residues linked via 1,4-glycosidic bonds, constitutes an architectural element in the cell walls of brown macroalgae. In brown algae, M-blocks (PolyM) are assembled first and then partially altered into MG-blocks (PolyMG) and G-blocks (PolyG) as the seaweed grows and adapts to the environment [1,2,3,4]. The stoichiometric distribution of these blocks governs physicochemical properties, with PolyG imparting calcium-dependent gel-forming capabilities and PolyM enhancing mechanical elasticity. However, its industrial applicability is constrained by elevated molecular weight and limited aqueous solubility. Thus, tailored depolymerization strategies are needed [3].

Alginate oligosaccharides (AOS) can be produced via chemical, physical, and enzymatic methods. Alginate lyases, catalogued within 17 polysaccharide lyase (PL) families in the CAZy database, catalyze the β-elimination reaction to cleave alginate chains, yielding bioactive AOS. While PL5, PL6, and PL7 families predominate in this enzymatic landscape, PL7 enzymes exhibit exceptional endolytic selectivity towards PolyM/PolyG substrates [5]. The resultant AOS exhibit bioactivity profiles intimately linked to their degree of polymerization (DP) and M/G stereochemistry. For instance, GGG oligomers suppress macrophage inflammatory responses by inhibiting NF-κB-mediated nitric oxide synthesis, while GG analogs disrupt bacterial membrane integrity to potentiate antibiotic efficacy [6,7,8,9]. Beyond therapeutic applications, AOS derivatives serve as elicitors of abiotic stress tolerance in crops, modulating antioxidative pathways and osmotic adjustment mechanisms [10].

However, their industrial application of alginate lyases was limited by intrinsic drawbacks, particularly poor thermostability. Many marine bacteria thrive at moderate-to-low temperatures, leading to alginate lyases that typically lose over 50% of activity after incubation at 40 °C for 30 min. The industrial production and application of AOS are significantly limited by its thermal sensitivity [11].

Recent protein engineering paradigms, including encompassing computational design, consensus sequence-guided mutagenesis, and scaffold remodeling, have yielded promising variants. Among them, the application of thermodynamic free energy ΔΔG is most common, which can assist in evaluating the mutation sites screened by multiple schemes and selecting the most stable ones. For instance, the PL-6 family brown algae lyase AlyRm6A first predicted stable mutation sites on FireProt web server and then obtained eight mutation sites through ΔΔG evaluation; the best one, Q216I, showed an increased half-life from 3.68 h to 4.54 in 50 °C and Tm from 61.5 °C to 63.5 °C [12]. The alginate lyase CelPL7A obtained single-point mutants with the same schedule of the FireProt web server and ΔΔG analysis, and the mutant G346S showed 3.2 times higher specific activity enhancement [13]. The alginate lyase VxAly7B-CM obtained 10 single-point mutants with computer-aided evolutionary coupling analysis and ∆∆G evaluation, and the Tm of E188N and S204G increased from 47.0 °C to 48.9 °C and 50.2 °C, respectively [14]. Jiang et al. identified lipase (Lipr27RCL) mutagenesis sites associated with enhanced flexibility based upon B-factor analysis and multiple sequence alignment, and then two mutated isoforms (Lipr27RCL-K64N and Lipr27RCL-K68T) were found to exhibit enhanced thermostability and improved residual activity [15]. Among all these means, B-factor analysis could locate highly flexible sites, and ΔΔG analysis could screen for most stable mutations. The combined use of B-factor and ΔΔG analysis could achieve a synergistic effect of “precise rigidity” and thermodynamic stabilization. It has significant advantages such as precise targets, low computational costs, easy connection with experiments, the ability to balance activity and stability, and explainable mechanisms. Notable successes include Q246V/K249V double mutants of AlyMc, which demonstrate a 63% extension in thermostability [16]. Also, the FlAlyA mutant H176D obtained by the same protocol was verified to have a Tm increase of 1.2 °C and a 50 °C half-life increase of 7.58 times [17].

Alyw203 was proven as a typical alkaline alginate lyase in our previous study [18] with industrial potential. However, this enzyme undergoes severe thermostability loss at temperatures exceeding 40 °C. To address the bottleneck of poor thermal stability in Alyw203, mutations were performed based on free energy and rigidity calculations. For the thermostability-enhanced mutants, molecular dynamics (MD) were conducted to investigate the molecular mechanism. The goal of this research is to overcome the poor thermal stability of alginate lyases using computer-aided strategies

## 2. Results and Discussion

### 2.1. Construction of the Alyw203 Mutants

**Figure 1:**
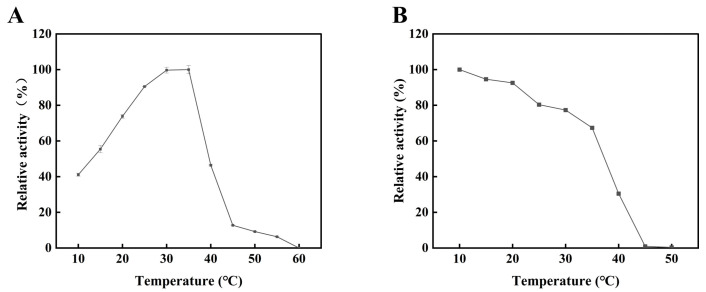
Effects of temperature on the activity (**A**) and stability (**B**) of Alyw203. The 100% relative enzyme activity in subfigures (**A**,**B**) is equal to 1163 U/mg.

The alginate lyase Alyw203 was recombinantly expressed in *E. coli* (Figure S1) with an enzymatic activity of 1338 U/mg and characterized as a typical cold-adapted enzyme. As illustrated in Figure 1A, Alyw203 exhibits an optimal reaction temperature of 35 °C. After 1 h incubation, Alyw203 retains maximal enzymatic activity at 10 °C and maintains over 65% residual activity within the 10–35 °C range. However, its relative activity sharply decreases to 30.46% at 40 °C, and it was completely inactivated beyond 45 °C (Figure 1B). Alyw203 shares similar properties with other cold-adapted alginate lyases in its family. AlyPM, a PL7 family alginate lyase, showed the best catalytic activity at 30 °C and retained high thermal stability below 35 °C [6]. Nevertheless, the poor thermostability of Alyw203 poses challenges in enzymatic production of AOS, reducing catalytic efficiency and complicating downstream product separation.

**Figure 2:**
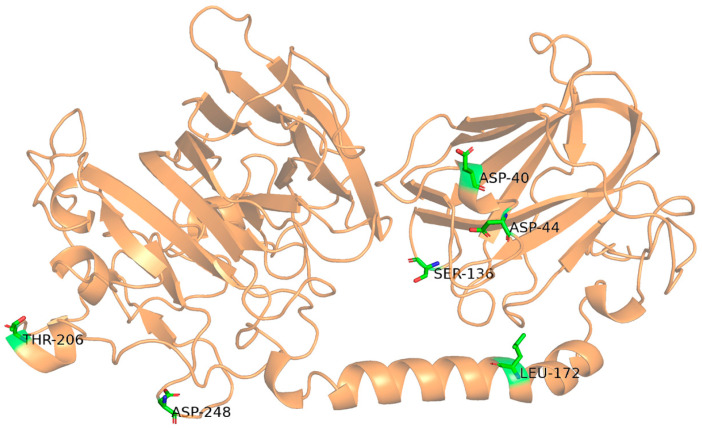
Schematic diagram of spatial location of mutation sites. Residues Thr206 and Leu172 are located at α-helical regions, while Asp248, Asp40, Asp44, and Ser136 are located at loop regions.

To address the poor thermostability of Alyw203, a mutagenesis strategy was employed to construct mutants of Aly203. Previous sequence alignment studies identified three conserved PL7 family motifs in Alyw203: “QIH,” “RTELREMLR,” and “MYFKAG” [11]. Homology modeling confirmed that Alyw203 adopts a β-sheet-dominated fold with minimal α-helical content, a structural hallmark of PL7 alginate lyases (Figure 2). AlphaFold 3.0 was used for refined structural modeling, and HotSpot Wizard 3.0 was used for computational hotspot analysis with B-factor and PoPMuSiC for ΔΔG values. The B-factor value reflects the “ambiguity” of the atomic conformation. The higher this value, the greater conformational fluctuation and the more unstable the structure of corresponding amino acid residue. Such highly flexible regions are often the key to affecting the thermal stability and other functions of proteins. ΔΔG values represent the difference in thermodynamic free energy between the mutant and the wild-type protein. Generally, negative ΔΔG shows that the mutant is more stable than the wild-type one. The electrostatic term represents the situation of the core energy contributing to ΔΔG. A negative electrostatic term indicates that this mutation mainly enhances protein stability by increasing electrostatic attraction (such as introducing complementary charges). When the electrostatic term is 0 or positive, it indicates that the protein stability obtained by this mutation is generated by other energy terms such as van der Waals forces, hydrogen bonds, and solvation. Then, the mutated amino acids of Alyw203 were selected based on criteria of high B-factor values (>22) and negative ΔΔG values (<0). These sites included four in loop regions and two in α-helical regions (Figure 2 and Table S1), all located outside conserved catalytic motifs. Following recombinant expression in *E. coli*, single-point mutants (D40W, D44I, S136I, L172V, T206W, D248L) were purified via Ni-column affinity chromatography. SDS-PAGE (Figure S2) confirmed successful purification, with all mutants displaying a prominent band at ~53 kDa, matching the molecular weight of wild-type Alyw203.

Notably, residues with high B-factor values or evolutionary variability often correlate with thermal liability, guiding prioritization for mutagenesis [19,20]. The rational selection of amino acid residues for improving thermostability in alginate lyases relies on a nuanced understanding of structural flexibility, catalytic dynamics, and evolutionary conservation. As demonstrated across multiple studies, residues located in regions of high conformational flexibility are prime targets for mutagenesis.

### 2.2. Enhanced Thermostability of the Mutant L172V

**Figure 3:**
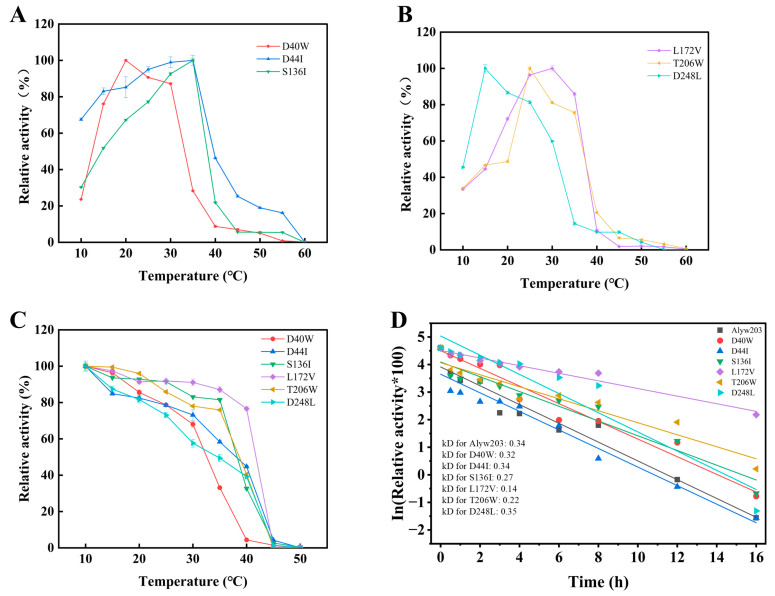
Effects of temperature on activity and stability of different mutants. (**A**) Optimal reaction temperature of mutants D40W, D44I, and S136I. (**B**) Optimal reaction temperature of mutants L172V, T206W, and D248L. (**C**) Thermal stability of different mutants by incubating enzymes at 10–50 °C for 1 h. (**D**) Half-life curves fitted by incubation of Alyw203 and its mutants at 40 °C for different times. The 100% relative enzyme activity for Alyw203, D40W, D44I, S136I, L172V, T206W, and D248L is equal to 1163, 1105, 784, 1512, 1318, 1309, and 1182 U/mg, respectively.

As shown in Figure 3A,B, the optimal temperatures of mutants, S136I, L172V, T206W, and D44I were consistent with that of Alyw203, maintaining high activity between 20–35 °C. However, D40W and D248L retained no more than 30% of the activity at 35 °C. As shown in Figure 3C, the mutants D40W, D44I, and D248L displayed lower relative activities than Alyw203 after incubation at 35 °C for 1 h, indicating reduced thermal stability. In contrast, mutants S136I, L172V, and T206W retained over 75% relative activity. After incubation at 35 °C for 1 h, the relative activities of S136I and T206W were 32.71% and 40.43%, respectively, showing slight but not significant improvements in thermal stability compared to Alyw203. Notably, the relative activity of L172V increased from 30.46% to 76.59%, demonstrating a substantial enhancement in thermal stability.

Further analysis of enzyme half-lives was conducted by incubating purified enzyme solutions at 40 °C for varying durations. As shown in Figure 3D, after 3 h incubation at 40 °C, Alyw203 was nearly inactivated, with a half-life (t_1_/_2_) of 122 min, indicating limited thermal stability. The half-lives of the mutants S136I and T206W were 154 min and 129 min, slightly higher than that of Alyw203 but with no significant improvement in thermal stability. In contrast, the half-life of L172V at 40 °C increased to 297 min, which was 2.43 times that of Alyw203. Moreover, L172V retained 37.80% of the activity after 12 h of incubation at 40 °C. Comparing their deactivation rate constants at 40 °C (Table S2), it can be concluded that L172V has the smallest kd value, indicating that it is the slowest to inactivate at 40 °C. These results indicate that the L172V mutation not only preserves the high stability and activity of the original enzyme at low temperatures but also significantly enhances its thermostability.

This study did not explore the thermal stability of Alyw203 at higher temperatures, as 35–40 °C is a reasonable temperature in industrial AOS production by the degradation of alginate with alginate lyase, taking into account the enzymatic hydrolysis efficiency, product quality, process economy, and stability. Within this temperature range, the water solubility of the alginate substrate fully meets the production conditions, and the production operation is safer. At the same time, it reduces the thermal degradation and color change caused by high temperature, also with lower energy consumption and production cost. In addition, product control can be better achieved at low temperatures. Therefore, the improvement in enzyme stability at 40 °C also has practical production significance.

Modifications targeting the flexible regions of alginate lyase have yielded progress in improving its thermostability. Rational design methods, such as ΔΔG calculations and B-factor analysis, improve stability by optimizing hydrogen bonding and structural rigidity, achieving 1.5–1.6-fold increases in the half-life of AlyMc [16]. Molecular dynamics simulations further refine the stability of ALYI1 by identifying flexible regions and stabilizing substrate interactions [20]. Site-directed mutagenesis targeting structural flexibility, exemplified by S72Ya in AlyG2, reduces surface loop mobility, enhancing thermostability [7,20]. Domain truncation strategies improve the stability of PeAly15-DUF by removing destabilizing domains [21]. These findings underscore the importance of structure-guided mutagenesis in industrial enzyme engineering. The stability enhancement facilitates the cost-effective production of customized AOS for applications ranging from precision medicine to circular biomanufacturing.

### 2.3. Other Enzymatic Properties of L172V

**Figure 4:**
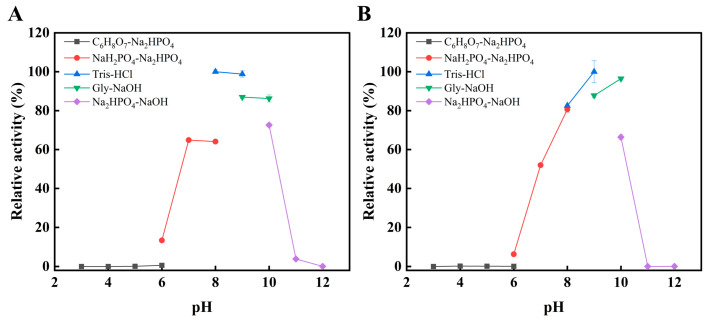
Effects of pH on activity of Alyw203 (**A**) and L172V (**B**). Their optimal pHs were determined in 10 mM buffers of varying pH [Na_2_HPO_4_-citric acid (pH 3.0–6.0), NaH_2_PO_4_-Na_2_HPO_4_ (pH 6.0–8.0), Tris-HCl (pH 8.0–9.0), glycine-NaOH (pH 9.0–10.0), and Na_2_HPO_4_-NaOH (pH 10.0–12.0)]. The 100% relative enzyme activity for Alyw203 and L172V was equal to 1338 and 1474 U/mg, respectively.

As shown in Figure 4A,B, the optimal pH for wild-type Alyw203 was 8.0, whereas the mutant L172V exhibited a shifted optimal pH to 9.0. Despite this upward shift, both the wild-type and mutant enzymes retained high catalytic efficiency across a broad pH range (7.0–10.0). The mutant retained the neutral-to-slightly alkaline characteristics of the parent enzyme. Its slight alkaline preference is advantageous for industrial applications, including alginate degradation in brown algae processing, which typically employs alkaline conditions for dissolution and extraction [22]. This alkaline preference is, in fact, not uncommon among reported alginate lyases. For instance, AlgA from *Pseudomonas* sp. E03 [23], AlyPL6 from *Pedobacter hainanensis* NJ-02 [24], FsAlyPL6 from *Flammeovirga* sp. NJ-04 [25], OUC-ScCD6 from *Streptomyces coelicolor* A3(2) [26], and AlgH from *Marinimicrobium* sp. H1 [27] exhibit optimal reaction pH values of 8.0, 10.0, 9.0, 9.0, and 10.0, respectively.

**Figure 5:**
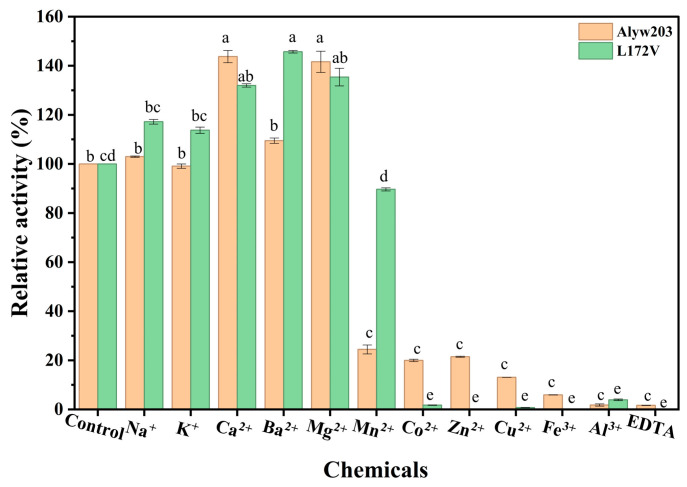
Effects of 1 mM of chemicals on activity toward alginate of Alyw203 and L172V. The 100% relative activity for Alyw203 and L172V was equal to 1338 and 1474 U/mg, respectively. Data points labeled with different lowercase letters are significantly different from each other (*p* < 0.05). The same letter indicates no significant difference. The data representing Alyw203 and L172V were subjected to significance analysis separately.

As shown in Figure 5, at 1 mM, Ca^2+^, Ba^2+^, and Mg^2+^ exerted varying degrees of positive effects on the activities of both wild-type Alyw203 and L172V. Ca^2+^ increased the relative enzyme activities of Alyw203 and L172V to 143.74% and 125.54%, respectively. Ba^2+^ demonstrated the most pronounced enhancement effect on the mutant L172V. However, other tested metal ions showed no significant activation effects, with some even inducing partial inactivation. In contrast, Co^2+^, Zn^2+^, and Mn^2+^ exhibited significant inhibitory effects on both enzymes. Notably, the inhibitory effect of 1 mM Mn^2+^ was attenuated in the mutant, with the residual activity increasing from 32.4% to 72.41%. Some reports showed that different alginate lyases can respond differently to metal ions. For example, while Mn^2+^ inhibits the alginate lyase from the alga *Pelvetia canaliculate* [28], it can activate some enzymes from bacteria [29,30]. In our wild-type enzyme Alyw203, Mn^2+^ most likely works as a competitive inhibitor. This hypothesis may be supported by the earlier observation that adding more substrate (PolyM) can reduce the inhibition caused by Mn^2+^ [28]. Our mutation L172V targets a residue in or near the predicted active site. This change might have disrupted a spot where cations bind or altered the local charge environment. As a result, the enzyme does not hold Mn^2+^ as tightly, so the competitive inhibition is much weaker at 1 mM. This explanation fits with the known importance of cation binding for the function of polysaccharide lyases [31]. To confirm this, we plan to perform detailed kinetic studies and structural analyses in the future.

### 2.4. Substrate Specificity and Kinetic Parameter Analysis

**Figure 6:**
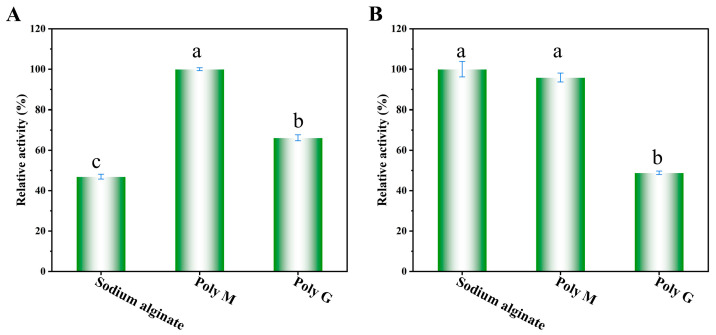
Substrate specificities of Alyw203 (**A**) and L172V (**B**). A total of 0.1 mL at 10 mmol/L of their optimal pH buffer containing 1% (*w/v*) substrates (sodium alginate, Poly M, and Poly G) was taken, 0.2 mL of enzymes (0.04 mg/mL) was added, and the mixture was reacted at their optimal reaction temperatures for 30 min. The 100% relative enzyme activity for Alyw203 and L172V was equal to 2854 and 1474 U/mg, respectively. Data points labeled with different lowercase letters are significantly different from each other (*p* < 0.05). The same letter indicates no significant difference.

As shown in Figure 6A, wild-type Alyw203 was a Poly-M-specific lyase. Further kinetic analyses for degrading different substrates also suggested that Alyw203 has the best substrate affinity and fastest catalytic efficiency for Poly-M (Table 1). As illustrated in Figure 6B, mutant L172V displayed elevated substrate specificity toward sodium alginate compared to Poly-M. Kinetic analyses of Alyw203 and L172V toward sodium alginate, performed across varying substrate concentrations (Table 1), revealed that L172V exhibited a reduced *K_m_* value (from 107 to 65 mg/mL) relative to the parental enzyme. Concurrently, its *k_cat_*/*K_m_* increased to 5-fold that of Alyw203 (from 0.07 to 0.35 mL/mg/s), indicating that the L172V mutation not only shifted substrate preference but also concomitantly enhanced substrate binding affinity and catalytic efficiency. Compared to another high-temperature-resistant PL7 family alginate lyase KAlLy (the value of *K*_m_ was 0.66 mg/mL) [32], the Alyw203 and L172V reported here have a higher Michaelis constant, indicating that its substrate affinity is much lower than KAlLy. Due to the different activity measurement methods used here, we cannot make a horizontal comparison of their catalytic constants. Compared with another PL7 family alginate lyase OalC6 using the same activity measurement method [33], the Alyw203 and L172V reported here also exhibited poorer substrate affinity and catalytic efficiency. These indicate that we need further efforts to enhance the substrate affinity and catalytic efficiency of the mutant. Of course, the semi rational modification approach validated in this article has positive reference significance for the modification of other alginate lyases.

Studies demonstrated that substrate preferences of alginate lyases can be reprogrammed via rational design or directed evolution. For instance, mutant Q246V of AlyMc exhibited enhanced polyG activity while preserving polyM specificity, whereas engineered Aly7Sa maintained strict G-specificity, exclusively generating unsaturated guluronate oligosaccharides from alginate [34]. The T91S mutation in AlyG2 elevated polyM degradation efficiency by 1.91-fold through reduced steric hindrance near the active site. Notably, thermostability-focused mutations, such as S72Ya in AlyG2, simultaneously enhanced thermal stability and polyM activity via loop rigidification [20]. These findings underscore the capacity to modulate substrate preference through active-site geometry optimization and binding-pocket adjustments.

### 2.5. Analysis of MD Simulations

**Figure 7:**
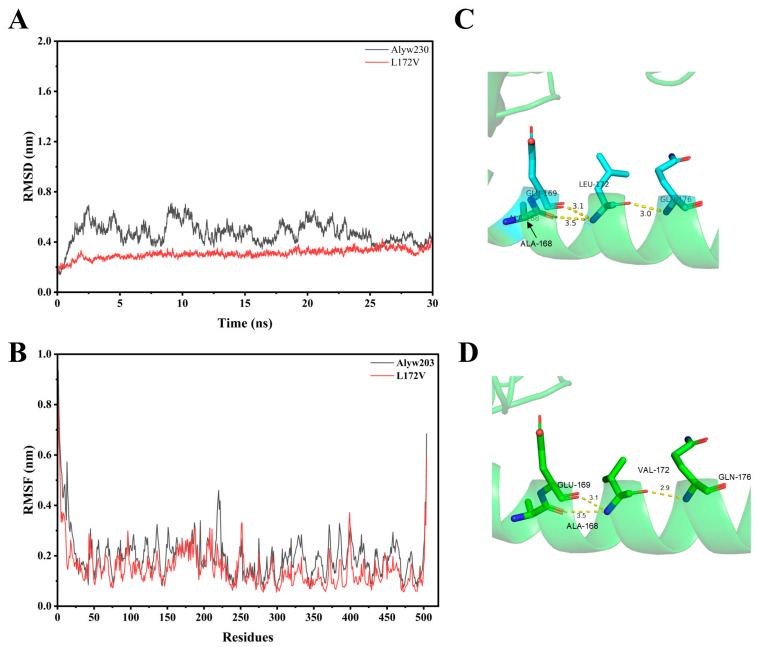
Comparison of root mean square deviation (RMSD), root mean square fluctuation (RMSF), and local hydrogen bonding network of Alyw203 and L172V. (**A**) RMSD of Alyw203 and L172V after a 30 ns molecular dynamics simulation; the average RMSD values of Alyw203 and L172V were 0.47 and 0.31, respectively. (**B**) RMSF of Alyw203 and L172V after a 30 ns molecular dynamics simulation; the average RMSF values of Alyw203 and L172V were 0.20 and 0.15, respectively. (**C**) The local hydrogen bonding network of Leu172 residue in Alyw203. (**D**) The local hydrogen bonding network of Val172 residue in L172V.

We leveraged 30 ns MD simulations at 300 K using Gromacs to evaluate the structural stability of Alyw203 and L172V mutants to check the root-mean-square deviation (RMSD). As shown in Figure 7A, the RMSD value of L172V was obviously lower than that of Alyw203. The average RMSD values of Alyw203 and L172V were 0.47 and 0.31, respectively. In addition, L172V reached equilibrium status within 5.0 ns, indicating that L172V was more rigid for better thermal stability. As shown in Figure 7B, the RMSF of residues 1−10, 210–220, 310–390, and 188−192 were lowered. It is widely accepted that lower RMSF values greatly contribute to thermal stability. The average RMSF of Alyw203 and L172V were 0.20 and 0.15, respectively. In conclusion, L172V had a highly rigid and more stable structure. By comparing the hydrogen bond networks around residue 172 in the wild-type enzyme and the L172V mutant (Figure 7C,D), it is evident that while no new hydrogen bonds were created by the mutation, the distance between residue 172 and residue 176 decreased by approximately 0.1 Å. This shortened hydrogen bond length corresponds to a stronger interaction, which helps to explain the enhanced activity (from 1338 and 1474 U/mg) and structural stability observed in the L172V mutant.

**Figure 8:**
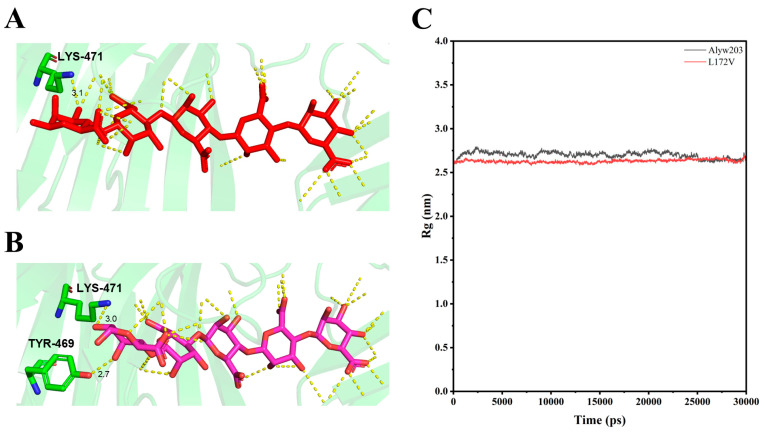
Comparison of substrate binding characteristics and radius of gyration (Rg) of Alyw203 and L172V. (**A**) Schematic diagram of hydrogen bonding between residue Lys471 and ligand in Alyw203–pentamannuronic acid. (**B**) Schematic diagram of hydrogen bonding between residues Lys471 and Tyr469 and ligand in L172V–pentamannuronic acid. (**C**) Rg of Alyw203 and L172V after a 30 ns molecular dynamics simulation; the average Rg values of Alyw203 and L172V were 2.70 and 2.63, respectively.

Molecular docking using AutoDock Vina 1.1.2 revealed distinct binding characteristics between Alyw203 and its mutant L172V with the substrate pentamannuronic acid (Figure 8A,B). In the L172V variant, a glycosidic bond at the substrate terminus exhibited significant torsional deflection, concomitant with the hydrogen bond between Tyr469 and the mannuronic acid residue, which stabilized the substrate conformation. Additionally, Trp139 established an additional hydrogen bond with the substrate, indicating enhanced binding affinity. These structural changes contributed to a reduction in the radius of gyration (Rg) of the enzyme–substrate complex; the average Rg values of Alyw203 and L172V were 2.70 and 2.63, respectively, reflecting increased compactness and conformational stability (Figure 8C). This enhanced structural integrity facilitates optimal substrate positioning and promotes efficient catalytic degradation, ultimately improving overall enzymatic efficiency.

The introduction of hydrogen bonds plays a pivotal role in modulating both the catalytic activity and structural stability of alginate lyases. In the engineered mutant AlyMc Q246V, the formation of additional hydrogen bonds around the mutation site contributed to enhanced structural rigidity and a 1.63-fold increase in thermal half-life [16]. Similarly, for the mutant mFsAly7, increased hydrogen bond occupancy resulted in reduced RMSF and improved thermostability, with a 4.4-fold longer half-life at 40 °C [35]. These findings underscore that hydrophobic interactions can minimize conformational flexibility and enhancing overall enzyme stability.

Regarding catalytic activity, hydrogen bonds directly influence substrate orientation and proton transfer mechanisms. In the case of VBAly15A, dynamic hydrogen bond interactions with key residues were essential for stable substrate binding at the -1 and +1 subsites. Disruption of these bonds led to a significant loss of activity, highlighting their role in positioning catalytic residues for efficient proton abstraction and glycosidic bond cleavage [36]. Furthermore, in Alg7A, hydrogen bonds between water molecules and substrate carboxyl groups optimized the electrostatic environment, facilitating syn β-elimination [35]. This synergy between hydrogen bonding and catalytic efficiency suggests that engineering strategies aimed at augmenting hydrogen bond networks can simultaneously enhance both stability and activity.

In addition, we observed that the three-dimensional structure of Alyw203 appears to differ from that of most reported alginate lyases in the PL7 family. For structural comparison with Alyw203, we selected the following PL7 alginate lyases: AlyA5 (GenBank: CAZ98266.1) and VxAly7D (GenBank: QPB15428.1) from *Zobellia galactinivorans* [37,38], TsAly7B (GenBank: QSG71221.1) from *Thalassomonas* sp. LD5 [39], and the crystallized PsAlg7C (PDB: 6YWF) from *Paradendryphiella salina* [40] for structural comparison with Alyw203 in this study. The structures of AlyA5, VxAly7D, and TsAly7B were predicted using AlphaFold3. As shown in Figure S3, AlyA5, VxAly7D, and PsAlg7C contain only the catalytic domain, whereas TsAly7B possesses two additional non-catalytic domains at its N-terminus. Structural alignment revealed that Alyw203 overlaps well with the catalytic domains of these enzymes in part. These results suggest that Alyw203 also contains an N-terminal non-catalytic domain (Figure S3E), the specific function of which requires further investigation through truncation experiments.

## 3. Materials and Methods

### 3.1. Strains and Chemicals

The marine bacterium *Vibrio* sp. B4 was isolated from abalone viscera and preserved in our laboratory. The pET-24a-Alyw203 plasmid was constructed in *E. coli*, and the cloning hosts *E. coli* DH5α and BL21(DE3) were ordered from Tsingke Biotechnology Co., Ltd. (Beijing, China). The Q5 site-directed mutagenesis kit was purchased from New England Biolabs Ltd. (Beijing, China). Genomic DNA extraction, plasmid extraction, and DNA gel recovery kits were obtained from Sangon Biotech Co., Ltd. (Shanghai, China). The DNS reagent was supplied by Solarbio Science & Technology Co., Ltd. (Beijing, China). The sodium alginate was obtained from Bright Moon Seaweed Group Co., Ltd. (Qingdao, China). The ploy M and ploy G were purchased from BZ oligo Biothch Co., Ltd. (Qingdao, China).

### 3.2. Design of the Aly203 Mutants

The full-length amino acid sequence of Alyw203 is provided in the Supplementary Materials. It shows 99% sequence similarity with a PL7 family protein from *Vibrio aphrogenes*, which was isolated from the surface of seaweed [41]. The three-dimensional structure of Alyw203 was predicted using AlphaFold. Potential mutation sites were identified based on B-factor analysis and ∆∆G values using the Hotspot Wizard 3.0 and PoPMuSiC web servers [16]. The mutation was performed using the Q5 kit with pET-24a-Alyw203 as a template, and the primers used for mutant construction are listed in Table S3. The PCR program included an initial denaturation at 98 °C for 30 s, followed by 25 cycles of 98 °C for 10 s, 50 °C for 30 s, and 72 °C for 210 s, with a final extension at 72 °C for 120 s. Positive clones were selected on LB agar plates containing kanamycin sulfate.

### 3.3. Protein Expression and Purification

The expression of recombinant wild-type Alyw203, along with its mutants, was conducted in BL21 (DE3) cells. The culture was transferred to fresh LB medium and grown at 37 °C with shaking at 180 rpm until OD_600_ reached 0.6. Protein expression was induced with 0.2 mM isopropyl β-D-thiogalactopyranoside (IPTG), followed by overnight incubation at 16 °C and 180 rpm. Following centrifugation at 8000 rpm for 10 min, the cell pellets were resuspended in 10 mL of phosphate buffer (pH 7.0). The resuspended cells were then subjected to 10 min of sonication and centrifuged again under the same conditions to collect the supernatant containing the crude enzyme. The enzyme was purified via nickel-affinity chromatography and analyzed using SDS-PAGE [16].

### 3.4. Alginate Lyase Activity Assay

The activity was assessed by using the DNS method. A total of 0.2 mL at 10 mmol/L and pH 8.0 of PB buffer containing 1% (*w*/*v*) sodium alginate was taken, 0.2 mL of AlyW203 (0.04 mg/mL) was added, and the mixture was reacted at 35 °C for 30 min. Then, 0.2 mL of DNS reagent was added and heated in boiling water for 5 min for color development. After cooling to room temperature in an ice water bath, distilled water was added to make up to 5 mL. The inactivated enzyme solution was used as a blank control group, and the absorbance value was measured at a wavelength of 520 nm. The generated reducing sugar content was calculated by substituting it into the glucuronic acid standard curve. The enzyme activity unit (U) is defined as the amount of enzyme required to generate 1 μg of reducing sugar per minute.

### 3.5. Thermal Stability Analysis

Enzyme activity was evaluated in 10 mM glycine–NaOH buffer (pH 8.0) at 10–60 °C to determine the optimal reaction temperature. Purified enzymes were incubated at 10–50 °C for 1 h, and then activity was measured at 35 °C. The activity of untreated enzyme was set as 100%, and residual activities were expressed as relative percentages. Additionally, enzymes were incubated at 40 °C for varying durations (0, 0.5, 1, 2, 3, 4, 6, 8, 12, and 16 h), and residual activity was determined. The half-life (T1/2) was calculated using the equation T1/2 = ln2/kd, where kd (inactivation rate constant) was derived from first-order regression using the equation lnA = kd × t (A represents residual activity, t represents incubation time).

### 3.6. Effect of pH and Metal Ions on Enzyme Activity and Stability

To assess pH effects, enzyme activity was measured in 10 mM buffers of varying pH [Na_2_HPO_4_-citric acid (pH 3.0–6.0), NaH_2_PO_4_-Na_2_HPO_4_ (pH 6.0–8.0), Tris-HCl (pH 8.0–9.0), glycine-NaOH (pH 9.0–10.0), and Na_2_HPO_4_-NaOH (pH 10.0–12.0)]. Reaction mixtures containing 0.1 mL of 2% (*w/v*) alginate and 0.2 mL of Alyw203 (0.04 mg/mL) were incubated at the optimal temperature for 30 min. Enzyme activity was determined via the DNS method, with the highest activity set as 100%, and the relative activity was calculated.

To assess the influence of metal ions and inhibitors, Alyw203 was incubated with each compound at final concentrations of 1 and 10 mM. A control reaction without additives was used to define 100% activity, and relative enzyme activities were calculated.

### 3.7. Kinetic Analysis

Kinetic characterization of Alyw203 and L172V was performed under the following conditions: 0.04 mg/mL of Alyw203 in 10 mM Tris-HCl buffer (pH 8.0) at 35 °C and 0.04 mg/mL L172V in 10 mM Tris-HCl buffer (pH 9.0) at 30 °C. Using substrate concentrations between 0.25 and 5.0% (*w*/*v*), reaction velocities were measured in triplicate and fitted to the Lineweaver–Burk model with the Origin 2021 software to obtain the kinetic parameters.

### 3.8. Substrate Docking Simulations

Homology modeling of Alyw203 and its mutants was conducted using AlphaFold 3.0. Substrate docking simulations with sodium alginate were performed using AutoDock Vina 1.1.2, and structural models were visualized using PyMOL 2.5.0 [42]. The comparison of the predicted structures of Alyw203 and L172V is presented in Figure S4.

Using Gromacs, molecular dynamics simulations were performed to analyze protein–ligand complexes over a 30 ns duration. The Amber99sb force field was selected, and the system was solvated with the single-point charge (SPC) water model in a periodic boundary box with a 1.0 nm distance. Appropriate amounts of sodium and chloride ions were added to neutralize the overall system charge. The system then underwent 50,000 steps of conjugate gradient energy minimization to stabilize the complex structure and eliminate possible steric clashes. This was followed by 200 ps of NVT ensemble equilibration and 200 ps of NPT ensemble equilibration. Finally, a 30 ns production molecular dynamics simulation was carried out under the isothermal–isobaric ensemble without constraints. After the simulation, the trajectory was analyzed by calculating the root mean square deviation (RMSD), root mean square fluctuation (RMSF), and the radius of gyration (Rg).

### 3.9. Data Processing

The experiment was performed in triplicate, and data were analyzed using Origin 2021. Microsoft Office 2019 was used for additional data processing. Statistical analyses were performed with SPSS 19 using Duncan’s test (*p* < 0.05) to check significant differences.

## 4. Conclusions

Rational design targeting flexible residues successfully enhanced alginate lyase thermostability. The L172V mutant exhibited a 2.43-fold longer half-life at 40 °C, reduced *K*_m_, and increased *k*_cat_/*K*_m_, indicating superior stability and catalytic efficiency. Molecular dynamics simulations revealed that these improvements originated from a reconstructed hydrogen bond network and reduced conformational flexibility, which stabilized the enzyme–substrate complex. This study validates a computational strategy for the precision engineering of alginate lyases and provides a robust, industrially promising biocatalyst for the efficient production of alginate oligosaccharides.

## Figures and Tables

**Table 1:** Kinetic parameters of Alyw203 and L172V. Kinetic characterization of Alyw203 and L172V was performed under the following conditions: 0.04 mg/mL of Alyw203 in 10 mM Tris–HCl buffer (pH 8.0) at 35 °C and 0.04 mg/mL L172V in 10 mM Tris–HCl buffer (pH 9.0) at 30 °C. Substrate concentrations between 0.25 and 5.0% (*w*/*v*) were used.

Substrates	Enzymes	*K*_m_ (mg/mL)	*V*_max_ (μmol/min/mg)	*k*_cat_ (s^−1^)	*k*_cat_/*K*_m_ (mL/mg/s)
Alginate	Alyw203	107 ± 3.56 ^a^	8.10 ± 0.64 ^c^	7.45 ± 0.59 ^d^	0.07 ± 0.006 ^d^
PolyM	Alyw203	43.7 ± 1.26 ^c^	26.6 ± 0.48 ^b^	24.5 ± 0.44 ^b^	0.56 ± 0.019 ^a^
PolyG	Alyw203	112 ± 4.56 ^a^	29.8 ± 0.75 ^a^	27.4 ± 0.69 ^a^	0.24 ± 0.012 ^c^
Alginate	L172V	65.0 ± 2.01 ^b^	24.5 ± 0.34 ^b^	22.5 ± 0.31 ^c^	0.35 ± 0.012 ^b^

Note: Data points labeled with different lowercase letters are significantly different from each other (*p* < 0.05). The same letter indicates no significant difference.

## Data Availability

The original contributions presented in this study are included in the article/Supplementary Material. Further inquiries can be directed to the corresponding author.

## Supplementary Materials

The following supporting information can be downloaded at: https://www.mdpi.com/article/10.3390/md24010006/s1, Figure S1 SDS-PAGE protein electrophoretic analysis of the purified Alyw203. Lane M: marker and lanes 1: pure enzyme, line 2: penetration peak; Figure S2: SDS-PAGE protein electrophoretic analysis of the purified mutant enzymes. Lane M: marker and lanes 1−6: D40W, D44I, S136I, L172V, T206W, D248L; Figure S3: Comparison of the structure of Alyw203 with different reported PL7 family alginate lyases; Figure S4: Comparison of the structure of Alyw203 and L172V; Table S1: AlphaFold was used to compute the relevant parameters of simulated single mutants; Table S2 AlphaFold was used to compute the relevant parameters of simulated single mutants; Table S3: Primers used in this study.

## Abbreviations

The following abbreviations are used in this manuscript:

AOS	Alginate oligosaccharide
M	β-D-Mannuronate
PolyM	Homopolymeric M-blocks
DP	Degree of polymerization
MD	Molecular dynamic
IPTG	Isopropyl β-D-thiogalactopyranoside
RMSD	Root mean square deviation
RMSF	Root mean square fluctuation
Rg	Radius of gyration
