# Supplementary material for: Enhanced Thermostability and Catalytic Efficiency of Alginate Lyase Alyw203 by Hydrogen Bond Network Reconstruction
Source: Mar Drugs. 2025 Dec 22;24(1):6. doi: 10.3390/md24010006 (PMC12843337; doi:10.3390/md24010006)
Supplement: Supplementary file 1 [file marinedrugs-24-00006-s001.zip › marinedrugs-4009082-supplementary.pdf]

Supplementary Materials

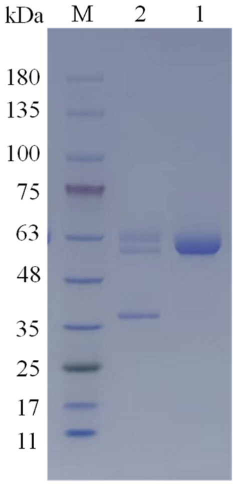

Figure. S1 SDS-PAGE protein electrophoretic analysis of the purified Alyw203. Lane M: marker and lanes 1: pure enzyme, line 2: penetration peak.

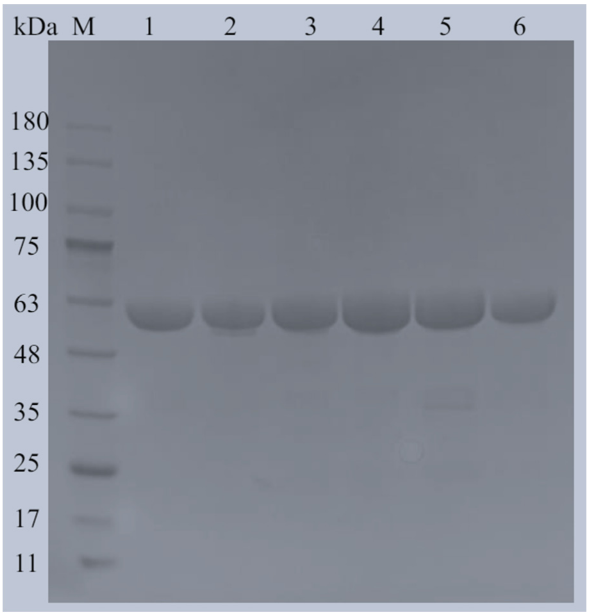

Figure. S2 SDS-PAGE protein electrophoretic analysis of the purified mutant enzymes. Lane M: marker and lanes 1−6: D40W、D44I、S136I、L172V、T206W、D248L.

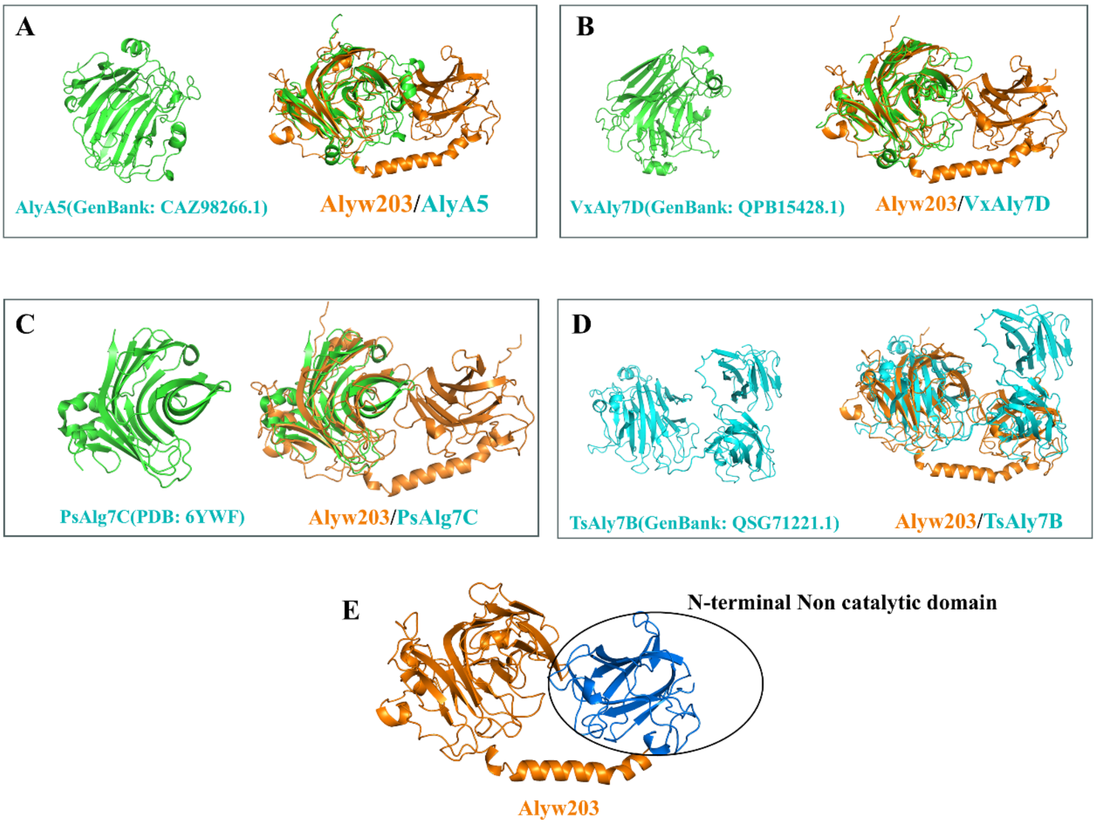

Figure. S3 Comparison of the structure of Alyw203 with different reported PL7 family alginate lyases. (A) The predicted structure of PL7 alginate lyase AlyA5 from *Zobellia galactanivoran*s and its overlap with structure of Alyw203. (B) The predicted structure of PL7 alginate lyase VxAly7D from *Zobellia galactanivoran*s and its overlap with structure of Alyw203. (C) The structure of PL7 alginate lyase PsAlg7C (PDB: 6YWF) from *Paradendryphiella salina* and its overlap with structure of Alyw203. (D) The predicted structure of PL7 alginate lyase TsAly7B from *Thalassomonas* sp. LD5 and its overlap with structure of Alyw203. (E) The predicted structure of Alyw203, where the blue part represents its N-terminal non catalytic domain. The predicted structures of AlyA5, VxAly7D, and TsAly7B were obtained using Alphafold3.

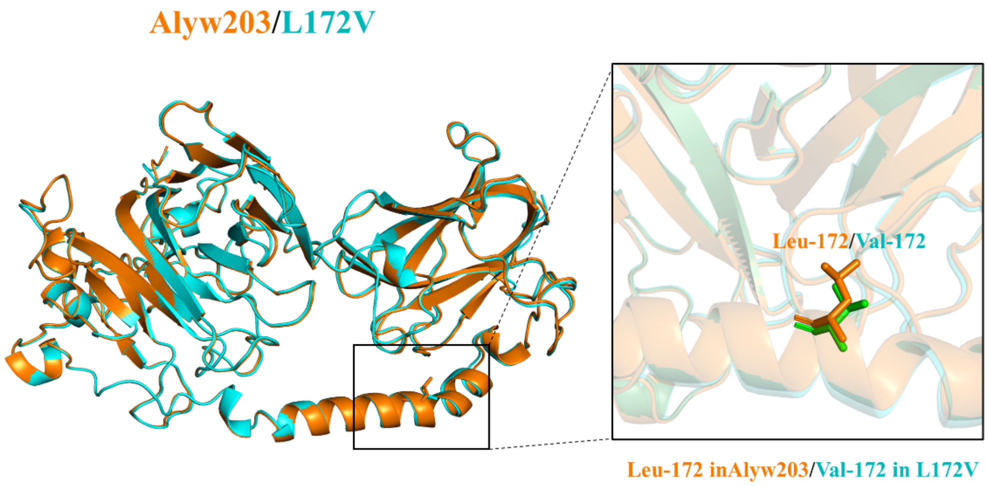

Figure. S4 Comparison of the structure of Alyw203 and L172V.

Table S1 AlphaFold computes the relevant parameters of simulated single mutants

Mutation	Mutation Energy	Effect of Mutation	Electrostatic Term
A:ASP40>TRP	-4.43	Stabilizing	-3.45
A:ASP44>ILE	-4.41	Stabilizing	-7.53
A:ASP248>LEU	-4.31	Stabilizing	-4.77
A:LEU172>VAL	-2.11	Stabilizing	-0.21
A:THR206>TRP	-1.22	Stabilizing	0.85
A:SER136>ILE	-1.13	Stabilizing	0.94

Table S2 AlphaFold computes the relevant parameters of simulated single mutants

Enzyme	Alyw203	D40W	D44I	S136I	L172V	T206W	D248L
kD(40°C)/ h^{-1}	0.34	0.32	0.34	0.27	0.14	0.22	0.35
$t_{1/2}$(40°C)/ min	122	130	122	154	297	129	119

Table S3 Primers used in this study.

primer name	Sequence (5′-3′)
Alyw203F	AAGGAGATATACATATGGTCGGTTGTACATCAAACGGGAATG
Alyw203R	GGTGGTGGTGCTCGAGTTTACCTTGATAGGTGCCGTGTGA
D40W-F	TAACGACCCTtggAGATTATTTGACCAAG
D40W-R	CCATCATGTGAGCTGGCT
D44I-F	TAGATTATTTattCAAGATATTAATACACGTTGG
D44I-R	TCAGGGTCGTTACC
S136I-F	TGGTAACACGattAATGGTTGGAAC
S136I-R	TGGCCGACATATTTTACG
L172V-F	CAGAGCAAGGtgtAATTGCACAGCAAAAAGAAG
L172V-R	CCGCAATGACAGCTGGAG
T206W-F	ATGTCAAACAtggGTTAAATGTGCCAAGACG
T206W-R	GGGTATACAGCTGGTACAC
D248L-F	ATTTGACCATctgAATAATAATCGCCC
D248L-R	GGCTGAGATAAATACCAAG

The full-length amino acid sequence of Alyw203, with the underlined portion being the signal peptide sequence:

MKHKIVKTLLASSVLFAVGCTSNGNDTSNLHPQSETGAPLLTPVAIEASSHD
GNDPDRLFDQDINTRWSANGDGEWAVLDYGSVHEFDAVRAAFSKGNERK
SKFDILVSTDGKTWTPVLQNQESSGGVIGYERFEFSPVQARYVKYVGHGNT
SNGWNSVTELAAVKCGVNACPSNQIITPAVIAAEQGLIAQQKEAEKARQA
ARKDLRKGNFGVPAVYPCQTTVKCAKTALPVPTGLPTTPKAGNKPSQNFD
LTSWYLSQPFDHDNNNRPDDVSEWDLANGYEHPDVFYTAKDGGLVFKSF

VKGVRTSPNTKYARTEMREMLRRGDTSIPTKGVNKNNWVFSSAPVADQK
AAGGVDGVMEATLKIDHTTTTGEAGEVGRFIIGQIHDQDDEPIRLYYRKLP
NHEKGTVYFAHENTLKGTDQYFDLVGGMTGEIGDDGIALGEKFSYRIAVK
GNTLTVTVMRDGKPDAKQVVDMSQSGYDVGGKYMYFKAGVYNQNITGE
MDDYVQATFYKLEKSHGTYQGK
